# Adult Onset Still's Disease: A Case Report with a Rare Clinical Manifestation and Pathophysiological Correlations

**DOI:** 10.1155/2013/981232

**Published:** 2013-01-17

**Authors:** Katerina M. Antoniou, George A. Margaritopoulos, Ioannis Giannarakis, Christianna Choulaki, Nikos Fountoulakis, Nikos M. Siafakas, Prodromos Sidiropoulos

**Affiliations:** ^1^Interstitial Lung Disease Unit, Thoracic Medicine Department, University Hospital of Heraklion, 71110 Heraklion, Crete, Greece; ^2^Department of Rheumatology, University Hospital of Heraklion, 71110 Heraklion, Crete, Greece

## Abstract

Adult-onset Still's disease is an inflammatory multisystemic disease of unknown etiology. Pleuritis is the most common pulmonary manifestation and pleural effusions are usually exudates with a predominance of neutrophils. We report a case of an eosinophilic pleural effusion as a novel and hitherto unrecognized manifestation of active adult-onset Still's disease. We also observed a marked NLRP3 inflammasome activation with increased production of IL-1**β** which coincided with the development and resolved upon remission of the pleural effusion suggesting a possible novel pathogenetic pathway for the development of pleural effusions in the context of the auto-inflammatory disorders.

## 1. Introduction

Adult onset Still's disease (AOSD) is a rare inflammatory disorder of unknown etiology characterized by high spiking fever, evanescent rash, arthritis, and multiorgan involvement [1]. The diagnosis that is one of exclusion and differential diagnosis includes infections, neoplastic, and autoimmune disorders. Although there are several sets of classification criteria, Yamaguchi's criteria [2] present the highest sensitivity (93.5%) (Table 1). Pleuritis is the most common pulmonary manifestation, and pleural effusions are usually exudated with a predominance of neutrophils [3].

Although AOSD pathogenesis is not known, it is considered as a member of the expanding group of the autoinflammatory disorders [4]. These diseases are caused by aberrancies in the innate inflammatory pathways, and increased release of active IL-1*β* is considered as a major event in their pathogenesis. Of note, in a small series of patients with AOSD, increased serum levels of proinflammatory cytokines (IL-6, IFN*γ*, IL-18, and IL-1*β*) have been reported [5]. NLRP3 inflammasome has been identified as the key intracellular platform for the maturation of IL-1*β*, and dysregulation of its function has been shown to have major role in the pathophysiology of autoinflammatory, metabolic, and certain autoimmune diseases [6]. While in systemic-onset juvenile arthritis (Still's disease) there is strong evidence supporting the role of innate immunity and IL-1*β* [7]; in AOSD the most strong finding suggestive of NLRP3 and IL-1*β* involvement is the clinical improvement with IL-1*β* inhibitors. We report a case of an eosinophilic pleural effusion as a previously unrecognized manifestation of AOSD associated with marked NLRP3 activation which resolved upon disease remission.

## 2. Case Presentation

**Figure 1:**
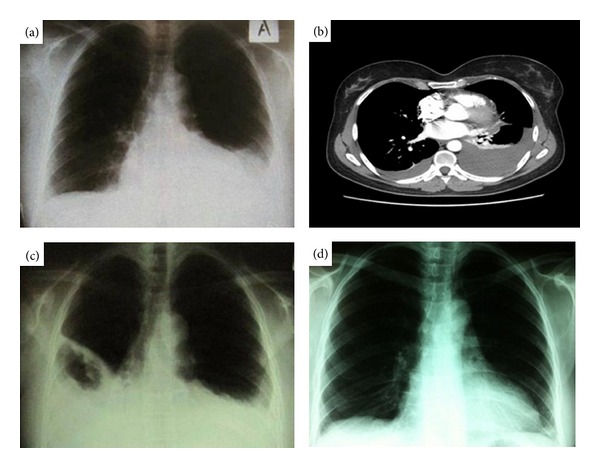
(a) Chest X-ray at presentation showing left-side pleural effusion. (b) CT pulmonary angiography showing bilateral pleural effusion. (c) Chest X-ray at the fifth day. (d) Chest X-ray at the third month.

 A 51-year-old never smoker woman who worked as a dentist presented to our department with a 15 days history of chest pain on the left hemithorax, mild shortness of breath on exertion, dry cough, evening fever (38.5°C), and diffuse arthralgias for the last 5 days. Both medical and family histories were unremarkable. On admission she was febrile (39.2°C) and tachypneic with a respiratory rate of 25 breaths/min. On physical examination there was decreased chest expansion, chest percussion and auscultation-revealed dullness, and reduced vesicular sounds in both lung bases, respectively. Arterial blood gases at rest showed a PH = 7.44, PO_2_ = 67 mmHg, and PCO_2_ = 29 mmHg. Laboratory findings included WBC count = 12000 cells/*μ*L, hemoglobin = 12.7 g/dL, and erythrocyte sedimentation rate (ESR) = 33 mm/h. Chest radiography revealed pleural effusion on the left lung (Figure 1(a)). Empiric treatment with broad spectrum antibiotics (ampicillin sulbactam and azithromycin) commenced immediately. Thoracentesis of the left-side effusion revealed an eosinophilic pleural effusion (EPE) (WBC = 5000, neutrophils = 38%, lymphocytes = 30%, and eosinophils = 20%). A work up for diagnosis of EPE was undertaken. Blood, urine, and sputum cultures, stains and cultures of bronchial wash obtained through fiberoptic bronchoscopy (FOB) and tuberculin skin testing were all negative for infectious causes. CT scan of chest and abdomen, ultrasound of abdomen, mammography, and FOB ruled out the presence of malignancy. CT pulmonary angiography was negative for pulmonary embolism but revealed a mild right pleural effusion as well (Figure 1(b)). Rheumatoid factor, antinuclear antibodies, antineutrophil cytoplasmic antibodies, and anticitrullinated protein antibodies were all negative. The patient denied any recent chest trauma or travel abroad and did not report any symptoms suggestive of parasitosis, whereas she was not on any drugs which could have caused EPE. On the fifth day of admission the patient became febrile again (39.4°C) with worsening of her breathlessness. A repeat chest X-ray revealed increase of the pleural effusion on the right side (Figure 1(c)), which required insertion of chest tube and removal of 1 liter of exudative fluid which also had characteristics of EPE (eosinophils = 22%), whereas stains, cultures, and cytology were all negative again. The patient kept complaining about arthralgias, and on rheumatologic examination a prominent arthritis of small joints of hands and feet as well as of large joints (knees) was detected. There was no evidence from her history and physical examination suggestive for a connective tissue disease, systemic vasculitis, or other chronic inflammatory disease. The most plausible diagnosis based on clinical and laboratory findings and exclusion of other possible causes of EPE was AOSD (total 4 Yamaguchi's criteria, 3 major). The patient was treated with corticosteroids, initially intravenously, and thereafter orally with improvement of her symptoms. Methotrexate was commenced as a steroid sparing agent, and she was discharged afebrile. Reevaluation after 3 and 6 months showed marked improvement of the radiological and clinical findings (Figure 1(d)). 

## 3. Discussion

AOSD is an inflammatory multisystemic disease of unknown etiology. Pleural effusion related to AODS is typically associated with predominance of neutrophils, and to our knowledge, this is the first report of EPE related to AOSD. According to a recently published study [8], the causes of EPE, include malignancies (mainly lung carcinoma), infections, medical or surgical procedures, transudative pleural effusions, spontaneous pneumothorax, pulmonary embolism, connective tissue disorders, and other less common causes. Malignancies are the most common cause (35%) of EPEs [8], while prevalence of malignancies between 1967 and 2009 has increased from 7% to 25% [9]. With our rigorous diagnostic algorithm we managed to exclude all the possible causes of EPE, and we therefore suggest for the first time that AOSD should be considered in the differential diagnosis of EPE. 

**Figure 2:**
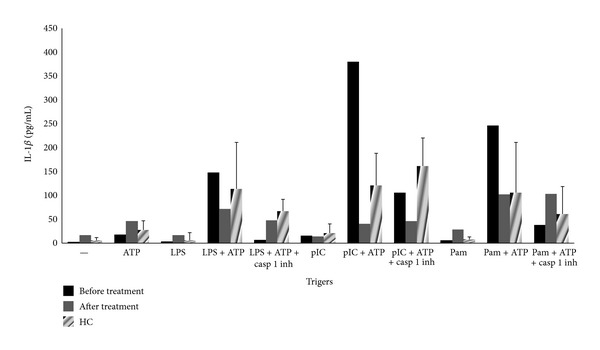
Active AOSD patients have increased NLRP3-mediated IL-1*β* production which is significantly decreased upon disease remission. Values are from AOSD case (at baseline and upon remission) and median (SD) from controls (*n* = 14). Values of IL-1*β* (pg/mL) were normalized according to white blood cell number per condition.

Since there are data supporting AOSD as an autoinflammatory disease, we sought to assess NLRP3-inflammasome activation and IL-1*β* production in our patient. Fourteen healthy adults were used as controls. Heparin-anticoagulated whole blood from AOSD case and controls (*n* = 14) was used after RBC lysis. LPS (250 pg/mL, 2 hrs), synthetic lipopeptide Pam_3_Cys (200 ng/mL, 2 h) and Poly(I : C) (50 *μ*g/mL, 2 h) were applied for TLR4, TLR2, and TLR3 stimulation, respectively. Specific NLRP3-inflammasome activation was followed with pulse ATP (5 mM, 20 min). Secreted IL-1*β* was assessed by Elisa. The caspase-1 inhibitor Ac-YVAD-CHO (10 *μΜ*) was used to inhibit caspase-1-mediated IL-1*β* production. At baseline (freshly isolated cells) minimal IL-1*β* production was found both in AOSD patient and controls. IL-1*β* secretion was significantly increased only after combined TLRs and NLRP3 stimulation (Figure 2). Active AOSD produced higher amounts of IL-1*β* upon combined TLR and NLRP3 stimulation, and the difference was even higher with TLR2 and TLR3 ligands. Interestingly, upon disease's remission within 3 months NLRP3-mediated IL-1*β* production was markedly decreased at levels comparable to or even lower (for TLR3 stimulation) than controls. Of note patients with NLRP3 mutations have shown to have a comparable pattern of IL-1*β* upon disease remission with IL-1Ra [10]. Another interesting finding that should be stressed is the increased IL-1*β* production upon PBMCs' priming with different TLR ligands, Poly(I : C) (TLR3 signaling) is included. This is interesting since TLR3 is involved in inflammatory responses upon viral infections and could explain AOSD flares upon viral infections.

To the best of our knowledge this is the first case to suggest that AOSD should be considered in the differential diagnosis of EPE and moreover to provide evidence of increased NLRP3-mediated IL1*β* production in active AOSD. Plainly the latter, although interesting, needs to be verified in a larger group of AOSD in order to support a pathogenetic role of NLRP3 inflammasome in AOSD. 

## Figures and Tables

**Table 1:** Yamaguchi's classification criteria.

Major criteria	Minor criteria	Exclusion criteria
(1) Fever >39°C intermittent for >1 week	(1) Sore throat	(1) Infections (2) Malignancies (3) Inflammatory diseases
(2) Arthralgia >2 weeks	(2) Lymphadenopathy and or slenomegaly	
(3) Typical rush	(3) Abnormal liver function	
(4) WBC > 10.000 (>80% granulocytes)	(4) RF, ANA: (−)	
